# Total joint reconstruction using computer-assisted surgery with stock prostheses for a patient with bilateral TMJ ankylosis

**DOI:** 10.1186/s40902-019-0225-1

**Published:** 2019-10-10

**Authors:** Seung-Hyun Rhee, Seung-Hak Baek, Sang-Hun Park, Jong-Cheol Kim, Chun-Gi Jeong, Jin-Young Choi

**Affiliations:** 1 Department of Oral and Maxillofacial Surgery, Seoul National University, Dental Hospital, Seoul, South Korea; 2 Department of Orthodontics, School of Dentistry, Dental Research Institute, Seoul National University, Seoul, Republic of South Korea; 3 Mir Dental Hospital, Daegu, Republic of South Korea; 4 FACEGIDE, Division of Digital Business, Megagen Implant, Daegu, Republic of South Korea; 5 Department of Oral and Maxillofacial Surgery, School of Dentistry, Dental Research Institute, Seoul National University, 101, Daehak-ro, Jongno-gu, Seoul, Republic of South Korea

**Keywords:** Total joint reconstruction, Recurrent TMJ ankylosis, 3D virtual surgical planning, CAD/CAM-fabricated surgical guides, Stock TMJ prostheses

## Abstract

**Backgrounds:**

The purpose of this study is to discuss the total joint reconstruction surgery for a patient with recurrent ankylosis in bilateral temporomandibular joints (TMJs) using three-dimensional (3D) virtual surgical planning, computer-aided manufacturing (CAD/CAM)-fabricated surgical guides, and stock TMJ prostheses.

**Case presentation:**

A 66-year-old female patient, who had a history of multiple TMJ surgeries, complained of severe difficulty in eating and trismus. The 3D virtual surgery was performed with a virtual surgery software (FACEGIDE, MegaGen implant, Daegu, South Korea). After confirmation of the location of the upper margin for resection of the root of the zygoma and the lower margin for resection of the ankylosed condyle, and the position of the fossa and condyle components of stock TMJ prosthesis (Biomet, Jacksonville, FL, USA), the surgical guides were fabricated with CAD/CAM technology. Under general anesthesia, osteotomy and placement of the stock TMJ prosthesis (Biomet) were carried out according to the surgical planning. At 2 months after the operation, the patient was able to open her mouth up to 30 mm without complication.

**Conclusion:**

For a patient who has recurrent ankylosis in bilateral TMJs, total joint reconstruction surgery using 3D virtual surgical planning, CAD/CAM-fabricated surgical guides, and stock TMJ prostheses may be an effective surgical treatment option.

## Introduction

Ankylosis of the temporomandibular joint (TMJ) is a disabling condition in mastication, speech, facial expression, pain, and oral hygiene, resulting in compromise of patient’s quality of life [1, 2]. The objectives in the treatment of TMJ ankylosis are to restore the masticatory function, improve the facial esthetics and phonation, relieve the pain, and prevent the re-ankylosis [3].

There are a variety of surgical options to manage the TMJ ankylosis including gap arthroplasty, interpositional arthroplasty, and total joint reconstruction [4]. In case of recurrent bony ankylosis, gap arthroplasty or interpositional arthroplasty has limitations to be applied due to low success rate and high relapse rate [5–7]. Total joint reconstruction can be performed with autogenous graft or alloplastic prosthetic device. Although costochondral graft has been considered the ‘gold standard’ for TMJ reconstruction of growing patients [8, 9], its outcome is reported to be unpredictable and often results in re-ankylosis of the TMJ [5, 7]. In this point of view, total joint reconstruction with alloplastic prosthetic device can be regarded as one of the reliable and effective surgical options for the end-stage TMJ ankylosis [10, 11].

The most frequently used alloplastic prosthetic device for total joint reconstruction is stock TMJ prosthesis and custom-made TMJ prosthesis [12]. The stock TMJ prosthesis system involves a two-stage protocol. During stage 1 surgery, the ankylotic bone is removed to create an adequate bony gap for placement of a spacer. After computed tomogram (CT) scan is taken, a stereolithographic model is fabricated to find the appropriate size and shape of stock TMJ prosthesis. At stage 2 surgery, the spacer is removed and the selected stock TMJ prosthesis is placed [3]. Its advantages are low-cost and no need for complex preparation for surgery.

On the other hand, the custom-made TMJ prosthesis system only requires a single operation. Due to use of computer-aided design and computer-aided manufacturing (CAD/CAM)-fabricated surgical guide and custom-made TMJ prosthesis, the precise operation can be performed with less time-consuming due to accurate match of the custom-made TMJ prosthesis with individual patient’s anatomy [13, 14]. Although it does not need a stereolithographic model, the custom-made TMJ prosthesis system has some disadvantages in terms of high cost and difficulty in obtaining government approval in some countries.

Therefore, it is necessary to combine the advantages of the two systems: low-cost and single operation with precise outcome. We combined three-dimensional (3D) virtual surgical planning, CAD/CAM-fabricated surgical guides, and stock TMJ prostheses for total joint reconstruction. The purpose of this case report was to discuss the total joint reconstruction surgery for a patient with recurrent ankylosis in bilateral TMJs using above-mentioned technology.

## Case report

**Fig. 1:**
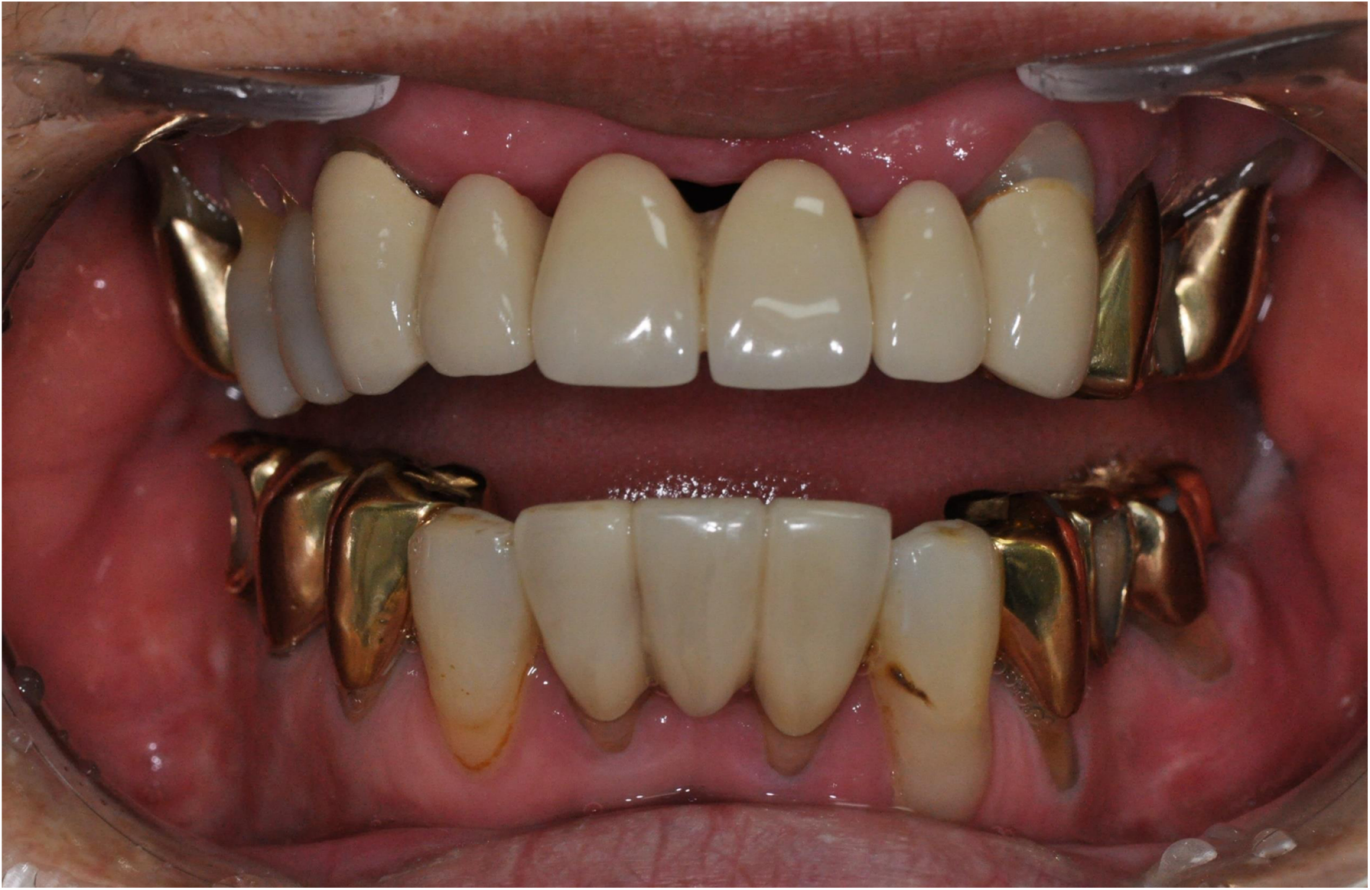
Intraoral frontal photograph of patient. The amount of maximum mouth opening was about 8 mm. The patient had a severe difficulty in eating due to limitation of jaw function and trismus

**Fig. 2:**
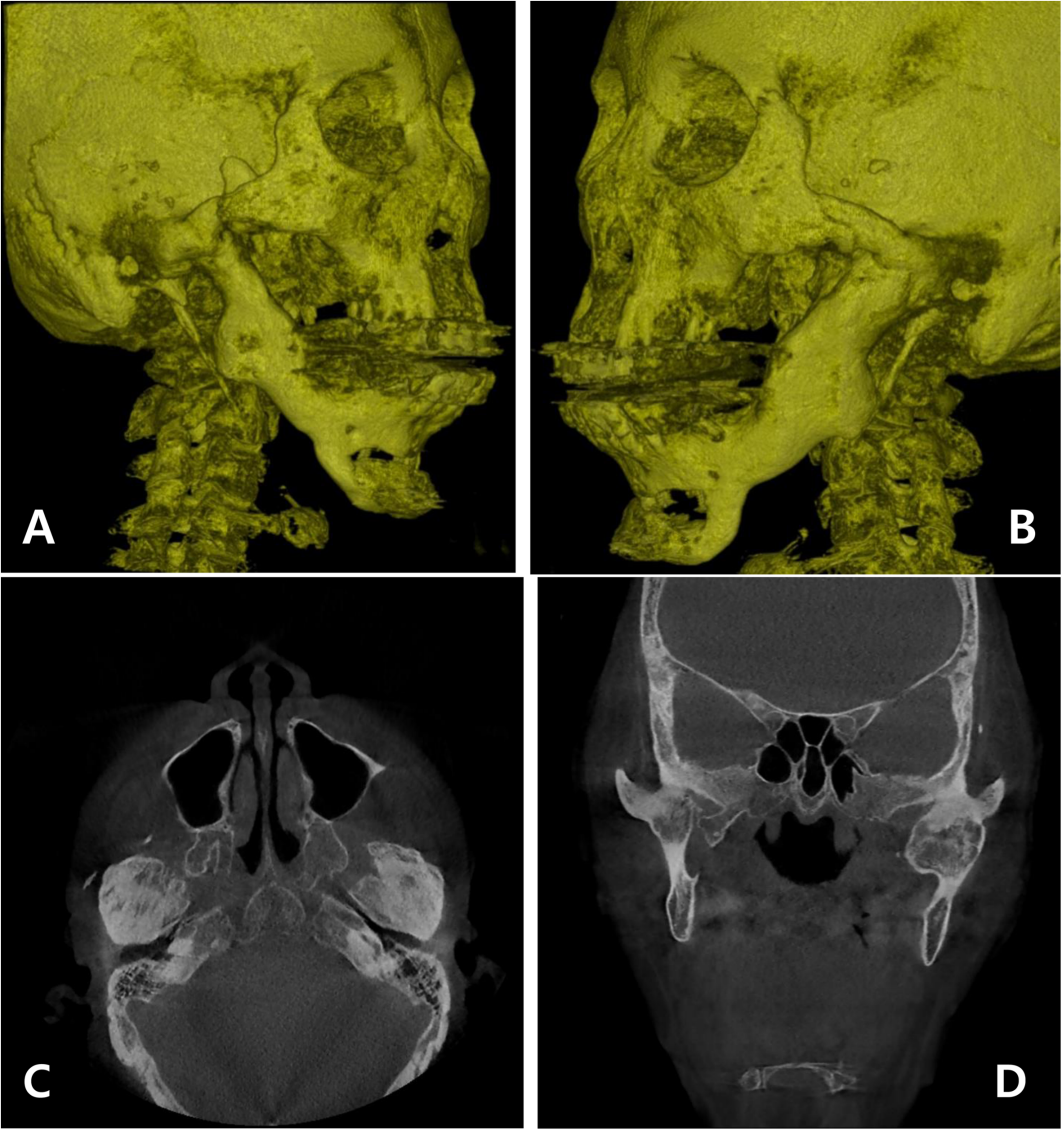
The computed tomogram (CT) images taken before total joint reconstruction surgery. Complete bony ankylosis of the temporomandibular joints (TMJs) at both sides was observed in the three-dimensional (3D) reorientation views (**a**, **b**), axial view (**c**), and coronal view (**d**)

A 66-year-old female patient visited the Department of Oral and Maxillofacial Surgery, Seoul National University Dental Hospital, with a complaint of severe difficulty in eating due to limitation of jaw function and trismus. The patient had a history of multiple TMJ surgeries for reduction of condyle fracture of both sides and arthroplasty/coronoidectomy due to trismus. Clinically, the patient exhibited the maximum mouth opening of 8 mm and relapse of severe trismus (Fig. 1). After CT scan was taken, type IV TMJ ankylosis on both sides was confirmed (Fig. 2). Since the glenoid fossa and condyle of both sides were completely fused, the anatomic structures were indistinguishable. Gap arthroplasty or interpositional arthroplasty could not be applied to this patient because bony TMJ ankylosis in both sides was complete and recurrent. Therefore, we decided to use the 3D virtual surgical planning, CAD/CAM-fabricated surgical guide, and stock TMJ prosthesis system (Biomet, Jacksonville, FL, USA) for total joint reconstruction.

The 3D virtual surgery was performed with a specific navigation surgery software (FACEGIDE, MegaGen implant, Daegu, South Korea). As the first step, both condyles and their surrounding hyperplastic bone were removed, while making it sure to avoid the mandibular foramen. The second step was virtual placement of the condyle and fossa components of the stock TMJ prosthesis. After placement of the fossa and condylar components, any areas of interference were investigated by virtually opening the jaw.

**Fig. 3:**
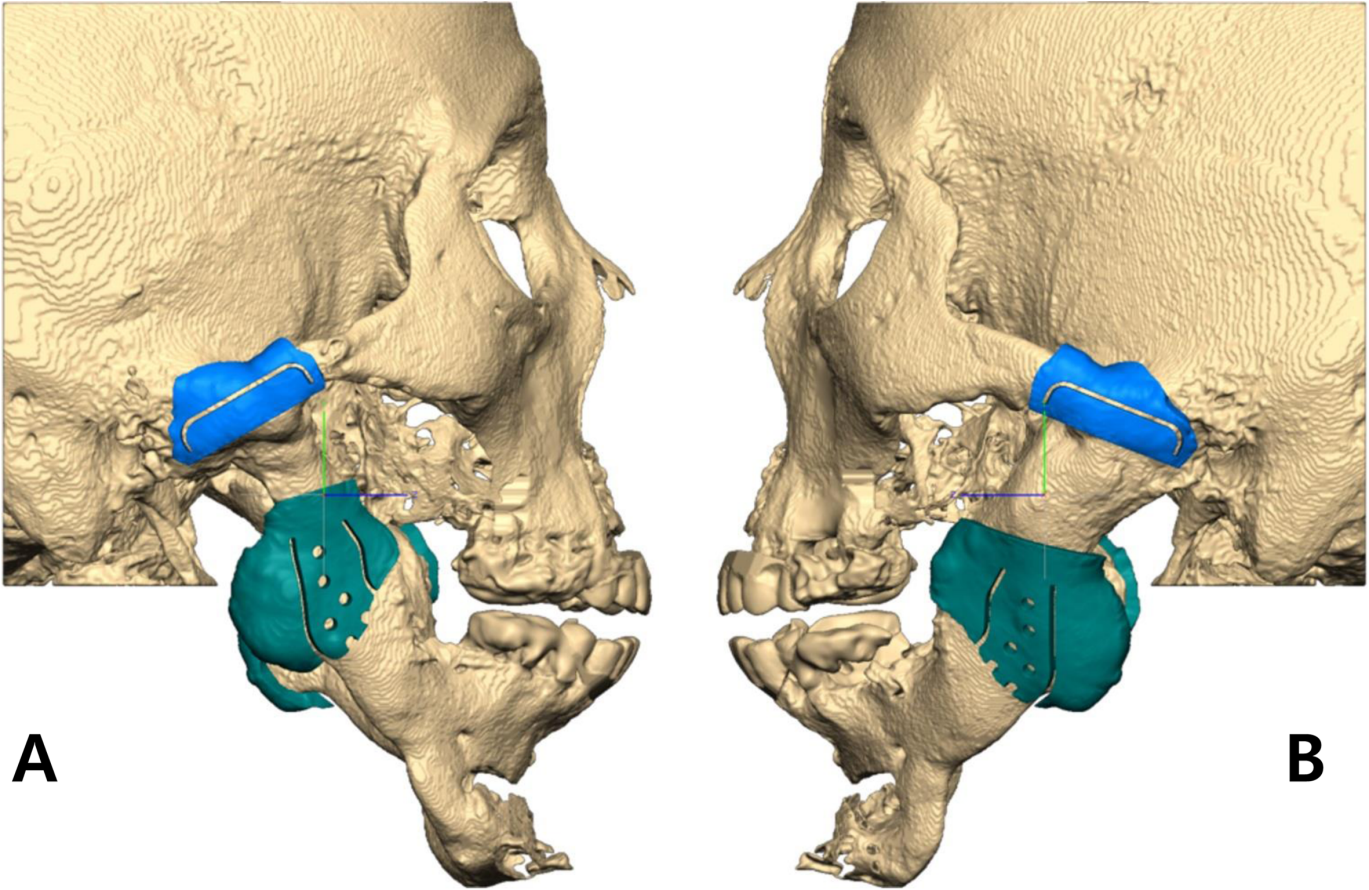
Right (**a**) and left (**b**) side of surgical guides using computer-aided design and computer-aided manufacturing. The upper part (blue) showed the location of upper margin for resection of the root of the zygoma and the location of the fossa component of stock TMJ prosthesis. The lower part (green) showed the location of lower margin for resection of the ankylosed condyle and several drill holes for screw fixation of the condyle component of stock TMJ prosthesis

After confirmation of the position of the fossa and condyle components, the surgical guides were fabricated by using the CAD/CAM technology. The surgical guides consist of the upper and lower parts. The upper part is designed to be adapted onto the root of the zygoma, which guides the location of upper margin for resection of the root of the zygoma and the position of the fossa component of the stock TMJ prosthesis. The lower part is designed to be adapted onto the mandibular angle area, which guides the location of lower margin for the resection of the ankylosed condyle and has several drill holes for fixation screws of the condyle component of the stock TMJ prosthesis (Fig. 3).

**Fig. 4:**
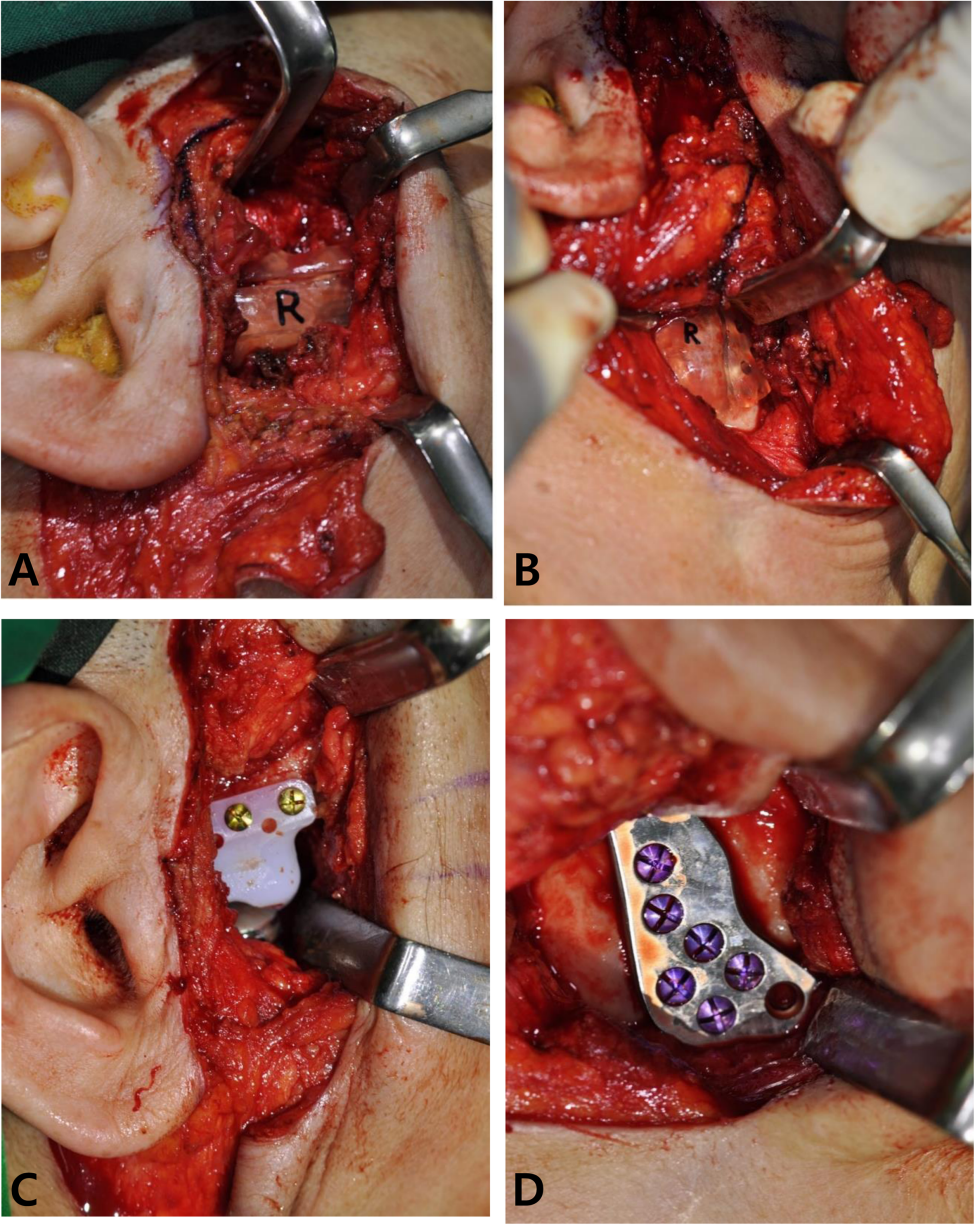
Intraoperative photographs taken at the right side. Placement of the surgical guide for the fossa component (the most left) and the condyle component (the second most left), and installation of the fossa component (the second most right) and the condyle component (the most right)

**Fig. 5:**
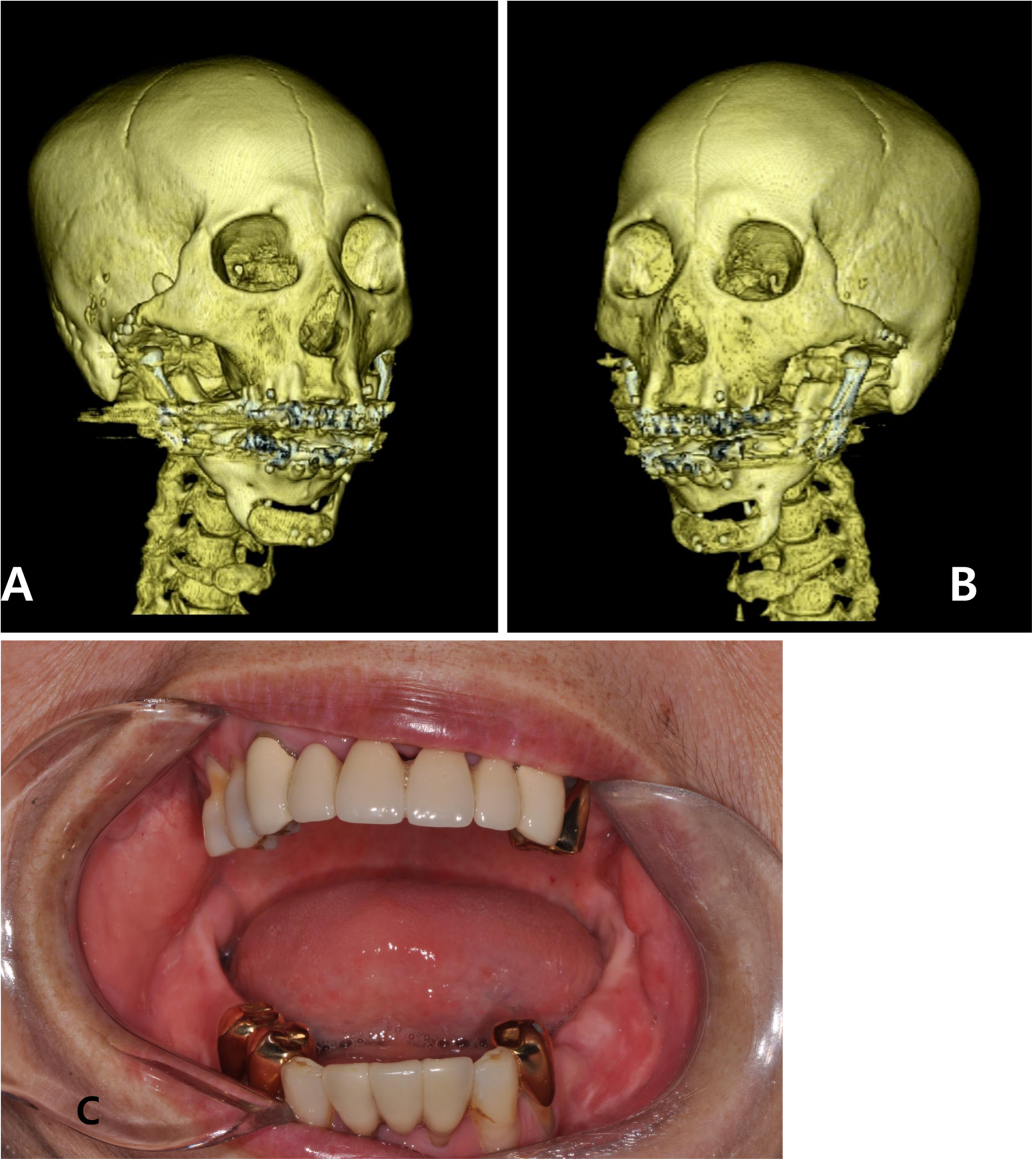
The 3D-CT images taken 2 months after surgery (**a**, **b**). Clinical photos of 2 months after surgery (**c**). The amount of maximum opening increases up to 30 mm

Under general anesthesia, the total joint reconstruction surgery was performed via preauricular and submandibular incisions. Osteotomy and placement of the stock TMJ prosthesis were carried out according to the 3D virtual surgical planning (Fig. 4). At 2 months post-operative follow-up (Fig. 5), the patient was able to open her mouth up to 30 mm without complication. She could undergo dental treatments that had been unavailable due to severe limitation of jaw function and trismus. One year after surgery of follow-up, the patient stated that she was living without any change of mouth opening range and side effects.

## Discussion

Complete bony ankylosis of the TMJ is a significant problem for patients as well as doctors. When the total joint reconstruction surgery with alloplastic materials is planned, it would be necessary to combine the advantages of stock TMJ prosthesis and custom-made TMJ prosthesis: a single-stage approach, low-cost, more precise surgical outcome, and less operation time-consuming.

There are several considerations for accurate placement of the stock TMJ prosthesis. First, since the stock TMJ prosthesis does not fit perfectly to the resection site due to straight design, leading to difficulty in properly positioning the condylar component medio-laterally. As a solution for this problem, the fossa component of the stock TMJ prosthesis system was fixated, followed by fixation of the condylar component and adjustment of its medio-lateral position by controlling the depth of the fixation screws.

Second, because the tissue surface of the condylar component of the stock TMJ prosthesis is flat, it is difficult to get a perfect adaptation onto the resection site. Therefore, the frontal ramus inclination of the condylar component of the stock TMJ prosthesis should be meticulously investigated. In the frontal view, if the lower margin of the condyle components of the stock TMJ prosthesis at the mandibular angle area is located medial to the glenoid fossa, the condyle head portion of the condyle components of the stock TMJ prosthesis may be positioned too laterally. In contrast, if the lower margin of the condyle components of the stock TMJ prosthesis at the mandibular angle area is located lateral to the glenoid fossa, opposite situation may arise. By controlling the insertion depth of fixation screws, such problems can be overcome. The insertion depth of fixation screws can be controlled while observing the relationship between the condyle head portion of the condyle component and the glenoid fossa. For example, if the frontal ramus inclination of the condylar component of the stock TMJ prosthesis is inclined to inward, the upper fixation screws for the condyle components can be inserted loosely, while the lower fixation screws can be inserted to their full depth.

The advantage of the system we used is that the cutting condyle and positioning TMJ prostheses can be determined according to the patient’s anatomy before surgery. Therefore, the need to perform the staged operation in two times is reduced, the operation time can be shortened, and using stock prostheses is cheaper than customized TMJ prostheses. In disadvantage, it’s less accurate than customized. The three-dimensional position can be grossly positioned the same as the surgical plan, but because the adaptation of prostheses is not accurate, minor differences such as the frontal ramal angle may occur. In order to overcome this drawback, the condyle prostheses should be placed first, and then the condylar part can be positioned more accurately. Another disadvantage of this system is that when condyle cutting, prediction of medio-lateral depth is not possible, so the perception of the safety zone toward the cranial base must be present before surgery.

In this case with recurrent bony TMJ ankylosis in both sides, all treatment objectives of total joint reconstruction surgery including improvement of mouth opening, correction of deformity, pain relief, and prevention of re-ankylosis were met with the use of 3D virtual surgical planning, CAD/CAM-fabricated surgical guides, and stock TMJ prostheses. However, it is necessary to increase the sample size and longitudinal long-term follow-up for evaluating possible complications.

## Conclusion

For a patient who has recurrent ankylosis in bilateral TMJs, total joint reconstruction surgery using 3D virtual surgical planning, CAD/CAM-fabricated surgical guides, and stock TMJ prostheses may be an effective surgical option to obtain precise surgical outcome with low-cost, less operation time consuming, and a single-stage approach.

## Data Availability

Not applicable.

## Abbreviations

3D: Three-dimensional
CAD: Computer-aided design
CAM: Computer-aided manufacturing
CT: Computed tomogram
TMJ: Temporomandibular joint
